# Spatial Omics Profiling of Treatment-Naïve Lung Adenocarcinoma with Brain Metastasis as the Initial Presentation

**DOI:** 10.3390/cancers17152529

**Published:** 2025-07-31

**Authors:** Seoyeon Gwon, Inju Cho, Jieun Lee, Seung Yun Lee, Kyue-Hee Choi, Tae-Jung Kim

**Affiliations:** Department of Hospital Pathology, Yeouido St. Mary’s Hospital, The Catholic University of Korea, Seoul 07345, Republic of Korea; 22580054@cmcnu.or.kr (S.G.); injucho81@gmail.com (I.C.); jieunlee@cmcnu.or.kr (J.L.); 22000657@cmcnu.or.kr (S.Y.L.); kuehee@cmcnu.or.kr (K.-H.C.)

**Keywords:** lung cancer, brain metastasis, digital spatial profiling, IDO-1, PD1, PDL1, STAT3, PTEN, CD44

## Abstract

Background/Objectives: Brain metastasis (BM) is a common and often early manifestation in lung adenocarcinoma (LUAD), yet its tumor microenvironment remains poorly defined at the time of initial diagnosis. This study aims to characterize early immune microenvironmental alterations in synchronous BM using spatial proteomic profiling. Methods: We performed digital spatial proteomic profiling using the NanoString GeoMx platform on formalin-fixed paraffin-embedded tissues from five treatment-naïve LUAD patients in whom BM was the initial presenting lesion. Paired primary lung and brain metastatic samples were analyzed across tumor and stromal compartments using 68 immune- and tumor-related protein markers. Results: Spatial profiling revealed distinct expression patterns between primary tumors and brain metastases. Immune regulatory proteins—including IDO-1, PD-1, PD-L1, STAT3, PTEN, and CD44—were significantly reduced in brain metastases (*p* < 0.01), whereas pS6, a marker of activation-induced T-cell death, was significantly upregulated (*p* < 0.01). These alterations were observed in both tumor and stromal regions, suggesting a more immunosuppressive and apoptotic microenvironment in brain lesions. Conclusions: This study provides one of the first spatially resolved proteomic characterizations of synchronous BM at initial LUAD diagnosis. Our findings highlight early immune escape mechanisms and suggest the need for site-specific immunotherapeutic strategies in patients with brain metastasis.

## Simple Summary

Brain metastasis is a serious and often early complication in lung adenocarcinoma, the most common type of lung cancer. While many studies have examined treated or advanced cases, little is known about the tumor environment at initial diagnosis. This study analyzed tissue from five patients whose brain metastases were the first signs of lung adenocarcinoma. Using spatial proteomic profiling, we compared protein expression between matched brain and lung tumors. Brain metastases showed reduced immune activity and increased markers of cell growth and immune evasion. These findings highlight the distinct biology of brain lesions even before treatment begins and suggest early immune escape. Understanding these early changes may support future efforts to develop site-specific strategies targeting brain metastases at initial presentation.

## 1. Introduction

Lung cancer remains one of the most frequently diagnosed malignancies and is the leading cause of cancer-related mortality worldwide, with approximately 2 million new cases and 1.76 million deaths annually [1]. Histologically, lung cancer is broadly classified into small cell lung cancer (SCLC) and non-small cell lung cancer (NSCLC), the latter accounting for approximately 85% of all cases [2]. NSCLC encompasses several subtypes, including squamous cell carcinoma, large cell carcinoma, and lung adenocarcinoma (LUAD), which is the most common histological subtype, representing approximately 40% of all lung cancers [3]. LUAD typically lacks specific symptoms in the early stages and is often diagnosed at an advanced stage. At the time of diagnosis, nearly two-thirds of patients present with stage IIIB or IV disease, characterized by a high propensity for metastasis and invasion into blood vessels, lymphatics, and nerves [4]. Consequently, the prognosis remains poor, with a reported 5-year overall survival (OS) rate of less than 20% [5]. Despite advances in early detection and treatment, the prognosis for patients with advanced-stage disease remains poor in many cases.

Brain metastasis (BM) is a frequent and clinically significant complication in LUAD. Approximately 30% of LUAD patients present with BM at diagnosis, and up to 50% will eventually develop BM over the course of their illness [6,7]. Unlike metastases from other primary tumors, BMs from lung cancer often occur within the first two years—or even concurrently—with the diagnosis of the primary tumor [8,9]. In some patients, BM is not only synchronous but serves as the initial presenting lesion that leads to the diagnosis of lung cancer. This clinical scenario is distinct and poses specific diagnostic and therapeutic challenges. A study from an Asian cohort showed that nearly one-third of such patients initially presented with neurological symptoms due to brain metastases, with a high rate of initial misdiagnosis and overall poor survival despite multimodal treatment [10].

Despite therapeutic advances, BM remains a major clinical challenge, with limited treatment efficacy and a median survival of only 3–6 months [8]. While various models have attempted to predict which patients are at high risk for BM [9], accurate early identification remains difficult. Moreover, as immune checkpoint inhibitors(ICIs) become standard in LUAD care, characterizing the tumor microenvironment (TME) of BM is increasingly important for informing treatment strategies.

The TME plays a central role in tumor progression, immune evasion, and resistance to therapy [11,12,13,14]. Specific microanatomic niches—including stromal regions [15,16], perivascular zones [17,18,19], and tumor–stroma interfaces [20,21]—have been shown to influence metastatic behavior. Most disseminated cancer cells fail to colonize secondary sites due to hostile microenvironments [22,23], and only a small subset with niche-modifying capabilities survive [24,25,26].

Recent multi-omic studies of matched LUAD and brain metastases have revealed substantial molecular divergence between sites and marked immune suppression within brain lesions [27]. These findings underscore the distinct biology of brain metastases. However, their spatial proteomic landscape—especially in treatment-naïve patients presenting with BM at initial diagnosis—remains poorly defined.

To address this gap, we applied digital spatial profiling (DSP) using the NanoString GeoMx platform to paired samples from five treatment-naïve LUAD patients, all of whom presented with BM as the initial clinical manifestation. This method enables high-plex, spatially resolved analysis of protein expression in formalin-fixed paraffin-embedded (FFPE) tissues while preserving tissue architecture [11,28,29,30]. By profiling immune- and tumor-related proteins across tumor and stromal compartments, our study provides insight into early immune escape mechanisms and site-specific features of metastatic adaptation before the influence of systemic therapy.

## 2. Material and Methods

### 2.1. Patients and Samples

Samples were obtained from the Department of Pathology at Yeouido St. Mary’s Hospital. Patients who underwent surgical resection or excisional biopsy for NSCLC between January 2014 and December 2019 were reviewed for inclusion. Among them, 34 patients had histologically confirmed NSCLC and had undergone resection of either the primary lung tumor, brain metastasis, or both. Of these, 18 patients met the criteria for synchronous brain metastasis as brain metastasis diagnosed within one month of the primary lung cancer diagnosis without a history of systemic or induction therapy prior to tissue acquisition [31]. Among the 18 synchronous cases, 17 were diagnosed with LUAD.

Within the 17 LUAD cases, 14 patients presented with brain metastasis as the initial clinical manifestation that led to the diagnosis of lung cancer. The remaining three patients were diagnosed with brain metastasis after the detection of the primary lung tumor. Among the 14 initial-presentation cases, 9 were excluded due to the absence or insufficient paired primary lung tumor tissue. Of the remaining seven with available paired samples, two were further excluded due to a history of systemic or induction therapy prior to tissue acquisition.

The final matched cohort included five treatment-naïve patients (three women and two men) with histologically confirmed primary pulmonary adenocarcinoma and synchronous brain metastases. In all five cases, brain metastasis was the initial clinical presentation, preceding the diagnosis of the primary lung tumor. This unique clinical context provided an opportunity to analyze the tumor microenvironment at the earliest phase of metastatic spread. All primary lung tumor samples were obtained via excisional biopsy. Formalin-fixed, paraffin-embedded (FFPE) whole-tissue sections from both primary lung tumors and brain metastases were prepared for digital spatial proteomic analysis. Clinical and pathological data were collected from electronic medical records and pathology reports. All samples were anonymized prior to analysis. The study was approved by the Institutional Review Board of Yeouido St. Mary’s Hospital (IRB No. 19SISI10155).

### 2.2. Digital Spatial Protein Profiling and ROI Selection

To detect proteins at high multiplex and spatial resolution, NanoString GeoMx^®^ Digital Spatial Profiling (DSP) technology (NanoString Technologies, Seattle, WA, USA) was employed to digitally quantify protein expression in formalin-fixed, paraffin-embedded (FFPE) samples. Whole-slide tissue sections (4 μm thickness) were prepared and incubated with a cocktail of UV-photocleavable oligonucleotide-conjugated antibodies.

A total of 95 circular regions of interest (ROIs; 100 μm in diameter) were manually annotated on digital slide images. ROI selection was guided by hematoxylin and eosin (H&E)-stained slides to capture four key anatomical compartments within the tumor microenvironment: tumor cell-rich area or stroma-rich area [32], perivascular stromal area [33], and tumor–stromal interface area [31]. For the evaluation of tumor microenvironmental heterogeneity, ROIs were classified into tumor-rich or stroma-rich regions based on the estimated tumor/stroma ratio. ROIs with ≥50% tumor cell content were defined as tumor-rich, and those with <50% tumor cell content as stroma-rich, in accordance with the cutoff proposed by Wu et al. [34], which has demonstrated prognostic significance across multiple solid tumors.

**Figure 1:**
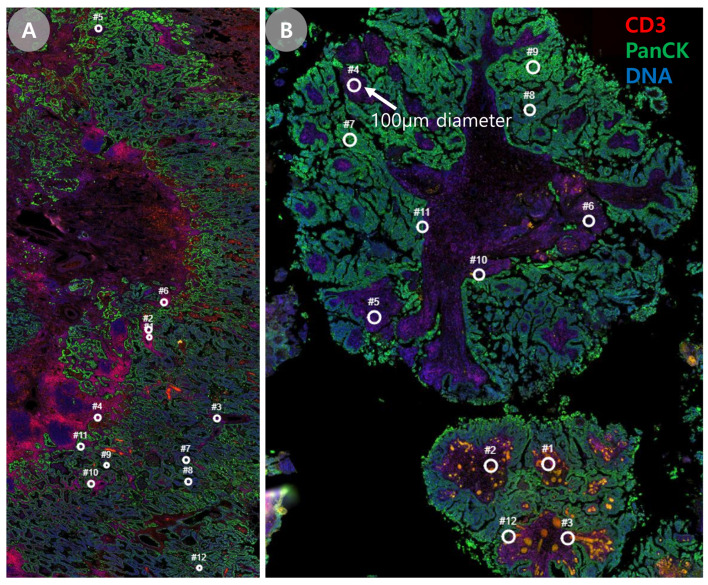
Representative images of DSP of primary lung cancer and brain metastasis. (**A**) DSP of primary lung cancer. FFPE tumor section was stained with fluorescent markers for CD3, PanCK, and DNA. Twelve ROIs were selected based on varying distributions of epithelium and immune cell expression. (**B**) DSP of brain metastasis. Likewise, 12 ROIs were selected in a single FFPE section of BM. After the exposure to UV light, spatially resolved oligos were collected and digitally counted for multiplex profiling via NanoString nCounter assay.

After UV illumination, oligonucleotides cleaved from the antibody panel were collected and analyzed via NanoString barcoding technology (NanoString Technologies). The assay included a commercial panel of 40 protein targets relevant to immuno-oncology, encompassing cell-type markers, immune checkpoints, signaling molecules, and controls. The profiled targets were as follows: AKT, B7-H3, B7-H4/VTCN1, Bcl-2, Beta-2-Microglobulin, Beta-Catenin, CD11c, CD14, CD3, CD4, CD44, CD45, CD45RO, CD56, CD66B, CD68, CD8A, FoxP3, GZMB, Histone H3, HLA-DR, ICOS/CD278, IDO-1, Ki67 (8D5), MmAb IgG2a, MS4A1 (CD20), P-AKT, Pan-Cytokeratin, PD-1, PD-L1, pS6, PTEN, Rabbit IgG, S6, STAT3, phospho-STAT3 (Y705), STING/TMEM173, and VISTA (Figure 1) [35].

### 2.3. Statistics

The paired *t*-test was used to analyze the difference between primary LUAD and brain metastasis. To document the differentially expressed proteins among ROIs, ANOVA Dunnett’s Test was applied. Statistical comparisons of protein expression among ROI subgroups (tumor cell, perivascular stroma, tumor–stromal interface, and stromal cell areas) were performed using one-way ANOVA followed by post hoc multiple-comparison correction. To control for the false discovery rate due to multiple testing across 40 protein targets, Benjamini–Hochberg correction was applied. Bonferroni correction was additionally considered for a highly conservative validation of significant findings. The analyses listed above and plots were performed using SPSS 21.0 (IBM, Armonk, NY, USA) and Rex (Version 3.0.3, RexSoft Inc., Seoul, Republic of Korea).

## 3. Results

### 3.1. Study Samples and Protein Profiling

Tumor tissues from five patients with primary LUAD and matched brain metastases were analyzed. A total of 120 ROIs were collected, encompassing both brain metastases and corresponding primary lung tumors. Protein expression profiling was performed using an oligo-conjugated antibody panel targeting 40 proteins, with quantification achieved through NanoString barcode readouts (Figure 1).

### 3.2. Differential Protein Expression Between Primary LUAD and Brain Metastases

**Figure 2:**
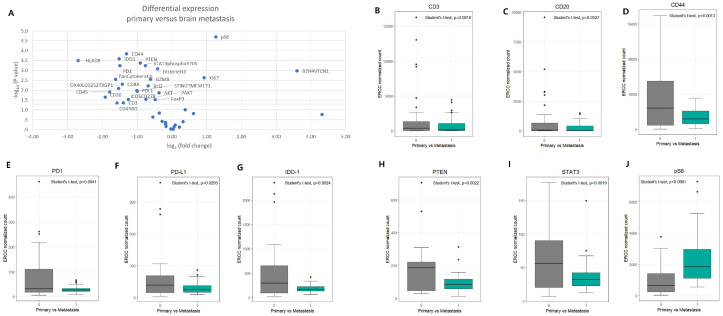
DSP analysis of primary lung cancer and brain metastasis. Protein profiling of ROIs was performed using oligo-conjugated panel and read out with NanoString barcodes. (**A**) Volcano plot showing differential expression of proteins between patients with primary lung cancer and brain metastasis. The x-axis shows the magnitude of a protein’s association with metastasis. The y-axis shows the −log10(*p* value), which increases with statistical significance. The horizontal blue line represents the *p* value cutoffs. Comparisons were analyzed using independent *t*-test. (**B**–**J**) Proteins associated with adaptive immunity and PTEN/mTOR/STAT3 signaling pathway documented lower expression in patients with brain metastasis. (**H**) However, protein expression pS6 was higher in brain metastasis.

DSP identified multiple proteins with significantly different expression between primary LUAD lesions and brain metastases (Figure 2A). Notably, CD3, CD20, CD44, IDO-1, PD1, PDL1, pS6, PTEN, and STAT3 showed statistically significant differences (*p* < 0.01). Proteins related to immune regulation and immune evasion—IDO-1, PD1, PDL1, STAT3, PTEN, and CD44—were expressed at lower levels in brain metastases (*p* < 0.01), whereas pS6, which is associated with activation-induced T-cell death, was significantly upregulated in metastatic lesions (*p* < 0.01) (Figure 2B–J and Table 1).

### 3.3. Histologic Distribution-Specific Protein Expression in ROI Subgroups

To assess spatial variation in protein expression, ROIs were subclassified into four anatomical compartments: tumor cell (TC), perivascular stroma (PVS), tumor–stromal interface (TSI), and stromal cell (SC) area.

**Figure 3:**
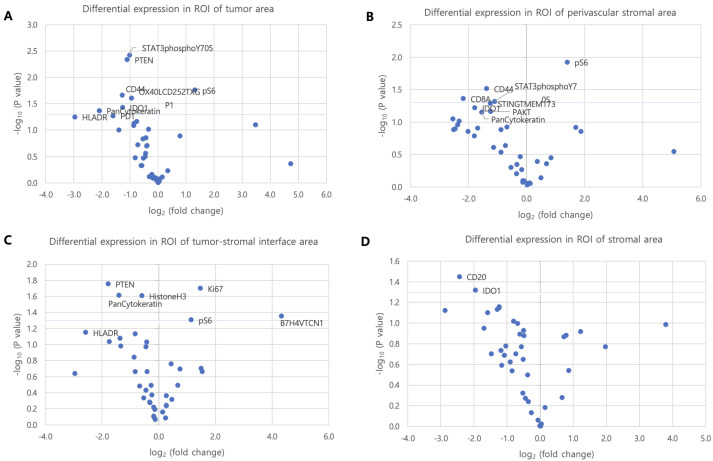
Volcano plot showing differential expression of proteins between patients with primary lung cancer and brain metastasis in ROI subgroups. The x-axis shows the magnitude of a protein’s association with metastasis. The y-axis shows the −log10(*p* value), which increases with statistical significance. The horizontal blue line represents the *p* value cutoffs. Comparisons were analyzed using independent *t*-tests. (**A**–**D**) Differentially expressed proteins were plotted according to the subgroups of ROIs.

In TC regions, significant differences were observed in STAT3, PTEN, CD44, IDO1, pS6, OX40LCD252TXGP1, and PanCytokeratin expression (all *p* < 0.05) (Figure 3A). PD1 and PDL1 exhibited a non-significant trend toward higher expression in primary lung cancer (*p* = 0.054 and *p* = 0.069, respectively). In PVS regions, CD44, CD8, pS6, and STAT3 were differentially expressed (*p* < 0.05), and IDO1 displayed a trend toward increased expression in primary lesions (*p* = 0.06) (Figure 3B). In TSI regions, PTEN, pS6, B7H4VTCN1, HistoneH3, Ki67, and PanCytokeratin exhibited significant differences (*p* < 0.05) (Figure 3C). In SC regions, CD20 and IDO1 levels were significantly higher in primary lung tumors compared to brain metastases (*p* = 0.04 and *p* = 0.05, respectively) (Figure 3D).

### 3.4. Intra-Group Comparison of Protein Distribution Within ROI Subtypes

**Figure 4:**
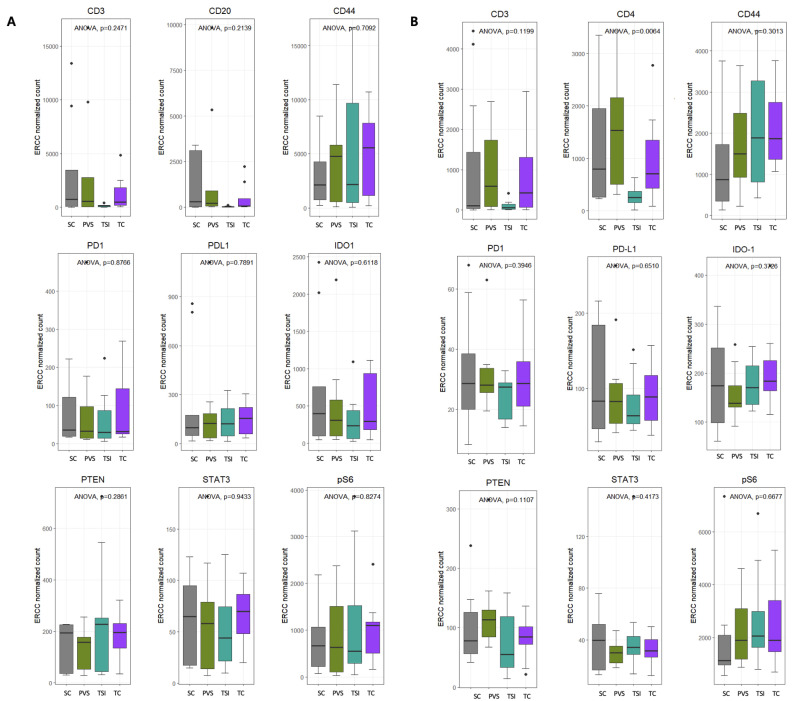
DSP expression in subgroups of ROIs in lung primary and brain metastasis. (**A**) In lung primary, there were no significant differences in the level of protein expression among ROI subgroups. (**B**) In brain metastasis, expression level of CD4 was higher in PVS with statistical significance (*p* value < 0.01).

Comparative analysis across ROI subgroups within each tumor type showed no significant intra-group variation in protein expression among primary lung tumors (Figure 4A). In contrast, in brain metastases, CD4 expression was significantly enriched in the PVS regions (*p* < 0.05) (Figure 4B).

### 3.5. Protein Expression According to Tumor–Stroma Composition

**Figure 5:**
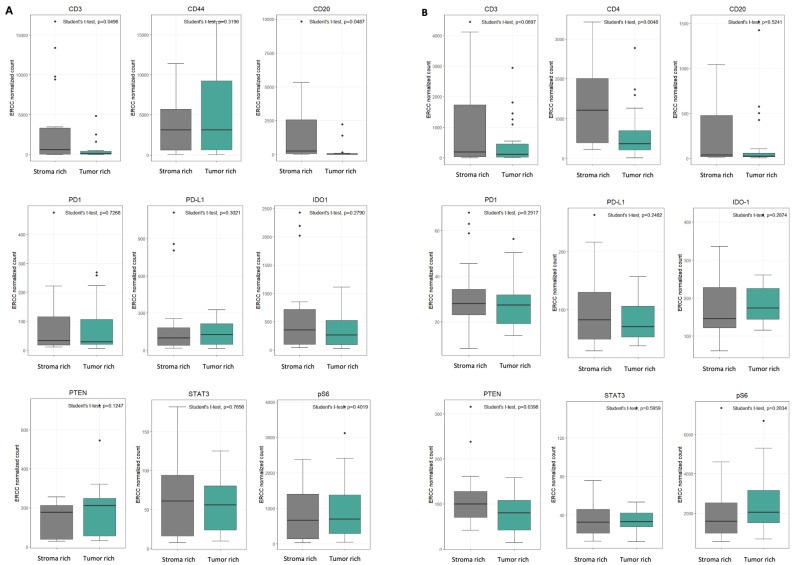
DSP expression in ROIs of tumor-rich subgroup and stroma-rich subgroup in lung primary and brain metastasis. (**A**) In lung primary, there were no significant differences in the level of protein expression among ROI subgroups. (**B**) In brain metastasis, the expression level of CD4 was higher in the stroma-rich area with statistical significance (*p* value < 0.01).

ROIs were further categorized into tumor-rich and stroma-rich areas based on a 50% tumor cell content threshold. In primary lung cancers, protein expression did not differ significantly between tumor-rich and stroma-rich regions (Figure 5A). However, in brain metastases, CD4 expression was significantly higher in stroma-rich areas (*p* < 0.01), suggesting the presence of an immune-enriched stromal microenvironment in metastatic sites (Figure 5B).

## 4. Discussion

Approximately 40% of patients with lung adenocarcinoma (LUAD) develop brain metastases (BMs) during the course of their disease [36,37]. BM is a poor prognostic factor, with untreated patients exhibiting a significantly worse median overall survival compared to those receiving treatment (4–11 weeks vs. 4–15 months) [38]. This study uniquely focuses on LUAD patients in whom BM was the initial clinical manifestation, providing insight into the tumor immune microenvironment prior to any systemic intervention.

Despite the high incidence of BM, patients with untreated or symptomatic brain metastases have been systematically excluded from major LUAD clinical trials of ICIs [39,40,41,42,43,44,45,46,47,48]. Exclusion criteria included the need for corticosteroids to control BM-related neurological symptoms and concerns regarding the limited ability of ICIs to cross the blood–tumor barrier due to their molecular size [49,50,51]. Furthermore, although cranial radiotherapy remains a standard treatment for BM, combining it with ICIs may enhance radiosensitization and elicit sustained immune responses—yet safety data on this combination remain limited [36].

Given the lack of studies in untreated BM, the biology of tumor-infiltrating immune cells in LUAD brain metastases warrants further investigation. Pedrosa et al. [9] attempted to identify molecular signatures predictive of BM, while Hung et al. [52] reported a significant association between the micropapillary histologic subtype and BM risk in LUAD. However, clinically validated biomarkers for stratifying BM risk remain elusive. In this study, we identified spatially and quantitatively distinct expression patterns of immune-related proteins between primary tumors and matched brain metastases. These findings may aid in the future identification of predictive biomarkers in LUAD patients with brain metastases.

The central nervous system (CNS) was thought to be an immune-privileged site. However, it has become evident that the CNS is immune-distinct rather than immune-privileged. It is now considered that the CNS undergoes constant immune surveillance, which takes place mainly within the meningeal compartment; furthermore, T-cells can cross the blood–brain barrier and blood–tumor barrier [36,53,54]. A unique aspect of our study is the evaluation of protein expression patterns across distinct tumor regions, including the TC, PVS, TSI, and SC areas. The differences observed between these compartments highlight the spatial heterogeneity of the tumor microenvironment.

While our study utilized manually selected ROIs based on pathologically defined compartments, this approach is inherently limited by interobserver variability and may fail to capture biologically informative regions not readily apparent through histology alone. To address these limitations, future studies could incorporate radiomics data in combination with deep learning-based algorithms to enable automated, unbiased, and reproducible ROI selection. Such an integrated radiology–pathology fusion strategy may provide a more comprehensive and representative spatial profiling of the tumor microenvironment.

The results of our study revealed that CD4+ T cells are differentially distributed among ROIs in brain metastases (*p* = 0.006), with significant enrichment observed in the perivascular stroma (PVS) region (Figure 4B). This spatial localization suggests that specific microenvironmental niches may serve as sites of active immune cell engagement or modulation. Notably, this enrichment may reflect an early mechanism of immune regulation or evasion, particularly within the perivascular niche. However, we did not distinguish among CD4+ T cell subsets—such as Th1, Th2, Th17, or FOXP3+ regulatory T cells (Tregs)—each of which can exert distinct and sometimes opposing immunologic effects within the tumor microenvironment [55]. Therefore, the possibility that immunosuppressive CD4+ subsets contribute to local immune evasion cannot be excluded. Immunohistochemical validation or additional multiplexed profiling using subset-specific markers (e.g., FOXP3, T-bet, GATA3, RORγt) [56] was not performed in this study, and caution is warranted in interpreting the biological significance of CD4+ T cell enrichment. Future studies incorporating high-dimensional single-cell or multiplexed spatial analyses will be essential to clarify the phenotypic identity and functional roles of CD4+ T cells in metastatic brain lesions.

Ribosomal protein S6 (rpS6) is one of the components of the 40S ribosomal subunit, and its phosphorylation is related to cell growth. Although not fully understood, rpS6 has been functionally regarded as the stimulator and/or inhibitor of cellular metabolism, cell size, survival, and proliferation [57,58,59]. Recently, several studies have explored the effects of rpS6 in tumors. Regulation of S6 phosphorylation and S6K activity is frequently altered in tumors such as lymphangioleiomyomatosis and renal carcinoma [60,61]. Moreover, several clinical trials are currently evaluating the antiproliferative efficacy of mTOR inhibitors, which inhibit the phosphorylation of S6 as treatment for human malignancies [62,63,64]. Kim et al. [65] evaluated the prognostic significance of S6 phosphorylation and its role in esophageal cancer. This study documented that high levels of pS6 were significantly associated with shortened disease-free survival and remained an independent adverse prognostic factor. Also, they demonstrated that the depletion of S6 and S6 kinase 1 resulted in a reduction in esophageal cancer cell migration and invasion [65]. Regarding the effect of pS6 LUAD, McDonald et al. [66] documented that pS6 is overexpressed in metastatic lung adenocarcinoma, and in primary tumors, higher pS6 expression is associated with shorter metastatic-free survival [66]. Also, Chen et al. [57] demonstrated that only hyperphosphorylation of rpS6 was significantly associated with the unfavorable survival and independent adverse prognostic value of patients with LUAD. Our study results revealed differentially expressed proteins regarding adaptive immunity. Among them, pS6 exhibited higher expression in brain metastasis (*p* < 0.0001). Within different ROIs in metastatic tumor, pS6 showed higher expression in the tumor-rich area rather than the stroma-rich area (*p* < 0.0004). However, no significant differences in the distribution among ROIs were observed in primary LUAD.

In our study, PD-1 and PD-L1 expression levels were lower in brain metastases compared to primary lung tumors. While previous meta-analyses have demonstrated clinical efficacy of PD-1/PD-L1 inhibitors in LUAD patients with brain metastases [67], it remains unclear whether this benefit is directly linked to PD1/PD-L1 expression within metastatic brain lesions. Our data do not permit a causal inference between local expression levels and treatment response, as the current study did not include clinical response data or functional immune profiling. Therefore, the observed expression differences should be interpreted with caution, and further investigation is warranted to determine whether systemic immune modulation or other factors account for the clinical benefit observed in these patients.

The decreased expression of IDO-1 in brain metastases observed in our study partially aligns with the findings of Chen et al. [68]. Their analysis revealed no significant difference in circulating IDO expression between patients with and without brain metastases, but they did identify cell type-specific expression differences. Specifically, the analysis of GEO datasets showed that IDO1 mRNA was highly expressed in myeloid cells in primary lung tumors, in natural killer cells in lymph node metastases, and in B cells in brain metastases. In our study, both CD20 and IDO1 levels in the SC compartment were significantly higher in primary lung tumors than in brain metastases. These findings suggest that immune suppression during the metastatic process may occur in a tissue-specific manner, and that mechanisms of immune evasion may differ depending on the metastatic site.

Also, our observation of reduced PTEN expression in brain metastases is consistent with the findings of Zhang et al. [69], who reported significantly lower PTEN expression in brain metastases compared to primary breast tumors and other metastatic sites. Their work further demonstrated that PTEN loss could be induced by the brain microenvironment and may be reversible, highlighting the dynamic influence of site-specific microenvironments on tumor behavior. Our findings suggest that a similar brain-mediated modulation of PTEN may occur in LUAD brain metastases.

Moreover, PTEN is a well-known negative regulator of the PI3K–AKT–mTOR signaling pathway, which governs cell survival, growth, and metabolism. Li et al. [70] proposed that genetic polymorphisms in this pathway are associated with an increased risk of brain metastasis in LUAD. Consistent with this, our study revealed decreased PTEN and increased pS6 expression in brain metastases, indicative of PI3K–AKT–mTOR pathway activation and enhanced pro-survival signaling.

Importantly, recent studies have also highlighted the role of PTEN in regulating tumor–immune interactions. PTEN loss has been associated with reduced antigen presentation, impaired T-cell infiltration, and resistance to immune checkpoint blockade—suggesting a dual role for PTEN in promoting both tumor growth and immune evasion [71,72,73,74]. Thus, the observed reduction in PTEN expression in LUAD brain metastases may contribute not only to metastatic outgrowth via enhanced survival signaling, but also to an immunosuppressive microenvironment that facilitates immune escape. These findings support the therapeutic relevance of PTEN-related pathways in both tumor-intrinsic and immune-mediated mechanisms of brain metastasis.

In addition, the decreased STAT3 expression observed in brain metastases in our study can be compared to the findings of Jin et al. [75], who reported that activation of the IL6/JAK2/STAT3 pathway in microglia induced an anti-inflammatory phenotype that promoted the progression of LUAD brain metastases. In our study, STAT3 expression was measured in tumor cells, suggesting that the observed decrease may reflect complex interactions between tumor cells and microglia. The proposal by Jin et al. [75] that the modulation of the IL6/JAK2/STAT3 pathway could be a promising strategy for suppressing brain metastasis adds therapeutic significance to our findings.

Although we observed reduced CD44 expression in brain metastases compared to primary lung tumors, this finding appears to contrast with previous studies reporting a critical role of CD44+ cancer-stem-cell (CSC)-derived pericyte-like cells in promoting brain metastasis [76]. One possible explanation is that CD44 positive cells may play a transient role during the early stages of metastatic colonization, with expression levels declining as the metastatic niche becomes established [77]. However, this interpretation remains speculative, as our study did not include temporal sampling or evaluation of additional pericyte markers (e.g., NG2, PDGFRβ) [78]. Thus, the role of CD44 in brain metastasis may be context-dependent and warrants further investigation using lineage tracing or time-resolved spatial profiling approaches.

To explain the observed discrepancy in CD44 expression between our findings and prior studies, we propose two non-mutually exclusive hypotheses, each with distinct biological implications.

First, CD44 may play a transient, stage-specific role during metastatic colonization. CD44 exists in multiple isoforms arising from alternative splicing, including the standard isoform (CD44s) and various variant isoforms (CD44v), which can exert contrasting effects on tumor progression. It is plausible that specific CD44v isoforms are transiently upregulated during the early phases of brain colonization to facilitate extravasation and niche establishment, but are subsequently downregulated once tumor cells adapt to the brain microenvironment. This isoform-specific transition could account for the reduced CD44 expression observed in our spatial proteomic data, which reflects established lesions. Future studies employing isoform-specific probes or RNA-based assays will be necessary to test this hypothesis.

Second, the reduced CD44 expression may reflect differences in cellular composition across the sampled compartments. While most prior studies focus on tumor-cell-intrinsic CD44 expression, CD44 is also expressed in stromal, immune, and pericyte-like cells. Our spatial profiling approach captures both tumor and non-tumor compartments, including perivascular and stromal regions. A decrease in CD44 may thus indicate depletion, phenotypic shifts, or reprogramming of CD44-expressing non-tumor cells in the metastatic niche, rather than changes in tumor cells per se.

If confirmed in larger datasets, these findings suggest that CD44 expression is not static but dynamically regulated during metastatic progression. The downregulation of CD44 in established brain metastases supports a “hit-and-run” model, in which CD44 expression facilitates early colonization but is later downregulated as tumor cells adapt or evade immune surveillance. This has therapeutic implications: anti-CD44 therapies may be more effective in preventing initial metastatic seeding than in treating established brain lesions. Moreover, brain-specific selective pressures may favor subclones with reduced CD44 expression, highlighting the need for context- and stage-specific therapeutic strategies.

To resolve these questions, future studies should employ isoform-specific antibodies and transcriptomic profiling to distinguish CD44s and CD44v across tumor and stromal compartments. In addition, lineage tracing and time-resolved spatial profiling will be essential and should be prioritized to map the dynamic regulation of CD44 during the metastatic cascade. These approaches will be critical in determining whether CD44 downregulation reflects adaptive reprogramming or clonal selection within the brain microenvironment.

Our observation of reduced IDO-1, PD-1, and PD-L1 expression in brain metastases aligns intriguingly with findings from recent matched miRNA profiling studies. For example, Tsakonas et al. [79] identified 11 differentially expressed miRNAs in matched primary NSCLC and brain metastasis samples, including a significant downregulation of miR-142-3p and miR-150-5p in brain lesions. Notably, miR-142-3p downregulation has been associated with the establishment of an immunosuppressive microenvironment, supporting our findings of reduced PD-1/PD-L1 and immune dampening in brain metastases.

A recent study has proposed miRNA signatures to predict brain metastasis. A 3-miRNA panel (miR-210, miR-214, and miR-15a) that predicted brain metastasis in LUAD patients with 90.4% accuracy [80]. Consistent with these findings, our study showed reduced expression of IDO-1 and STAT3—key regulators involved in shaping an immunosuppressive tumor microenvironment—which may also represent downstream consequences of miRNA-mediated adaptations that facilitate tumor survival in the brain metastatic niche [81].

The recent literature has highlighted the spatial heterogeneity of miRNA expression. Our study adds a distinct layer by analyzing anatomically defined compartments such as the perivascular stroma (PVS) and tumor–stromal interface (TSI). The significant increase in CD4 expression specifically within the perivascular region of brain metastases may imply active remodeling of the perivascular niche, potentially driven by miRNA-regulated mechanisms. This is in line with findings by Wang et al. [82], who showed that miR-596-3p modulates blood–brain barrier permeability through the YAP1–IL8 signaling axis.

Together, these findings suggest that brain metastasis involves a complex interplay between miRNA-regulated gene networks and proteomic remodeling. For instance, the upregulation of miR-378 has been shown to promote brain metastasis via MMP-2, MMP-9, and VEGF regulation [83], which complements our observation of elevated pS6 and suggests a pro-metastatic tumor microenvironment. These integrated insights underscore the potential of combining spatial proteomic profiling with miRNA analyses to uncover novel therapeutic targets and inform future research.

In summary, this study employs highly multiplex NanoString DSP to elucidate the immune context of brain metastasis in LUAD. Notable differences in the expression of immune-related proteins were identified between primary LUAD and brain metastasis, with lower levels observed for IDO-1, PD1, PDL1, STAT3, PTEN, and CD44 in brain metastasis, and higher expression of pS6. The spatial distribution of these differentially expressed proteins within distinct tumor microenvironment regions revealed a significant increase in CD4 expression in the perivascular stromal area of brain metastasis, challenging the traditional view of the central nervous system as an immune-privileged site.

Recent work by Qiu et al. [84] has characterized the proteomic features of small-sized but highly invasive LUAD, providing deeper insights into tumor aggressiveness and metastatic potential. The spatially resolved immune signatures identified in our study—despite being from metastatic lesions—are consistent with the notion that proteomic changes can reflect underlying biological behavior beyond tumor size, reinforcing the need for classification systems that incorporate both molecular and spatial parameters.

In terms of translational application, Gu et al. [85] have emphasized the utility of patient-derived xenograft (PDX) models in capturing tumor heterogeneity and evaluating therapeutic response in NSCLC. The brain metastasis-specific protein signatures we identified may be further validated using PDX models to assess their functional relevance and potential as therapeutic targets.

Additionally, Lu et al. [86] demonstrated the growing potential of nano-based immunotherapies in lung cancer. The immune-evasive features we observed in the brain metastatic microenvironment could inform the rational design of nano-immunotherapeutic strategies aimed at overcoming immune suppression in metastatic niches.

Together, these recent findings support the broader translational relevance of our study, while also highlighting future directions for functional validation and therapeutic development.

This study has several limitations. Given the relatively small sample size—comprising only five matched pairs of primary LUAD and brain metastases—this exploratory spatial proteomic study should be considered hypothesis-generating. The findings represent preliminary observations that require validation in larger, more diverse cohorts to ensure statistical robustness and generalizability. Given the extreme rarity of treatment-naïve LUAD patients presenting with brain metastasis at initial diagnosis, the acquisition of paired, high-quality FFPE tissues posed inherent structural constraints on sample size. Nevertheless, this unique clinical context offers a valuable opportunity to investigate early immune evasion mechanisms in brain metastasis.

While the limited cohort size is a clear constraint, it is important to note that prior studies using spatial proteomics or single-cell techniques in rare metastatic settings have demonstrated the value of small but biologically rich cohorts. For instance, small-cohort studies employing high-dimensional profiling have uncovered immune evasion signatures in leptomeningeal metastasis and adaptive resistance mechanisms in EGFR-mutant NSCLC [87,88]. These studies illustrate that hypothesis-generating insights can emerge from focused, high-resolution analyses, particularly when applied to clinically unique scenarios. Our study follows this precedent by leveraging spatial proteomics to investigate a rare and clinically informative cohort—treatment-naïve LUAD patients whose brain metastases were the initial manifestation of disease.

The inclusion of diverse spatial compartments (e.g., tumor core, stroma, and perivascular regions) and high-resolution profiling across 120 manually annotated ROIs allowed for an in-depth spatial characterization of the brain metastatic microenvironment, with biological replicates embedded in tissue architecture. Nonetheless, the limited cohort size warrants a cautious interpretation of the findings, and further validation in larger, independent cohorts will be essential to generalize these observations. Future efforts may benefit from multi-institutional collaboration or integration with public spatial transcriptomic repositories to increase statistical power and external reproducibility.

In addition, this study was conducted with a proteomics-centered approach, without transcriptomic profiling, which limits the opportunity for integrated multi-omics analysis and cross-platform validation. This constraint affects the interpretation of signaling dynamics and upstream transcriptional regulation.

The absence of an external validation cohort or accompanying functional assays also limits the ability to confirm the mechanistic or clinical relevance of the observed spatial protein expression patterns. As this is an exploratory single-cohort study, these findings should be considered hypothesis-generating and require functional corroboration in future research.

Although the ROIs were selected based on standardized pathological criteria to represent key anatomical and tumor compartments, the selection process remains manual and thus inherently subjective. This introduces the possibility of sampling bias and interobserver variability. Spatial heterogeneity across tumor subclones or immune niches may not be fully captured. Future studies could benefit from the incorporation of radiomics-guided or deep learning-based automated ROI selection, which could improve reproducibility, reduce human bias, and enable more comprehensive coverage of the tissue microenvironment.

Furthermore, while CD4+ T-cell enrichment was observed in some regions, we were unable to resolve functional subtypes (e.g., Th1, Th17, Treg) due to the limitations of the antibody panel. This restricts our ability to interpret the directionality of immune activation or suppression and highlights the need for expanded marker panels or multimodal platforms to enhance immune phenotyping.

While clinical outcome data were available, the small number of matched cases in this rare cohort precluded any statistically meaningful analysis of associations between spatial protein expression and patient prognosis or therapeutic response. Therefore, correlation analyses with clinical endpoints were not included in this study. Future studies with larger sample sizes will be necessary to enable such outcome-linked spatial profiling.

In addition, the cross-sectional design precludes the evaluation of temporal changes in protein expression during disease progression or treatment. A deeper understanding of the temporal evolution of the tumor immune microenvironment is critical to elucidating the biology of metastasis, especially during the early phases of niche formation and maintenance. Future studies incorporating longitudinal sampling and time-resolved spatial proteomic profiling will be essential to capture these dynamic shifts and clarify the timing and mechanisms of immune escape during metastatic progression.

Although digital spatial profiling (DSP) enables high-resolution proteomic analysis within preserved tissue architecture, it is inherently restricted to predefined ROIs. This limitation, combined with biological heterogeneity and potential technical variability in sample processing, may affect generalizability. Compared to unbiased whole-slide or transcriptome-scale spatial technologies, the ROI-based approach offers depth but limits breadth. These methodological considerations should be addressed in future studies employing larger cohorts, longitudinal sampling, and integrative multi-omics strategies.

## 5. Conclusions

This study highlights distinct immune alterations in brain metastases of LUAD using NanoString DSP. Key immune regulators—including PD1, PDL1, IDO-1, STAT3, PTEN, and CD44—were downregulated in brain metastases, while pS6 was upregulated. Notably, CD4+ cells were enriched in perivascular regions, suggesting spatial immune modulation. These findings provide insight into the unique tumor microenvironment of brain metastases and may inform future biomarker discovery and therapeutic strategies. Given the small sample size, these findings should be interpreted as preliminary. While they suggest potential avenues for biomarker discovery and therapeutic development, further validation in larger, independent cohorts will be essential to determine their biological and clinical relevance.

## Figures and Tables

**Table 1:** Differential expression of immune markers in brain metastases compared to primary LUAD across spatial compartments.

Marker	TC	TSI	PVS	SC	Relative Expression in BM
PD-1	↓	↓	↓	—	Down
PD-L1	↓	↓	↓	—	Down
IDO-1	↓	↓	↓	—	Down
STAT3	—	↓	↓	—	Down
PTEN	↓	—	—	—	Down
CD44	↓	↓	↓	↓	Down
pS6	↑	↑	↑↑	↑	Up (in PVS)
CD4	—	—	↑	—	Up (in PVS)

**Abbreviations:** TC, tumor cell; TSI, tumor–stroma interface; PVS, perivascular stroma; SC, stromal cells; BM, brain metastasis.

## Data Availability

Data are contained within the article.

## Abbreviations

The following abbreviations are used in this manuscript:

BM	brain metastasis
CNS	central nervous system
CSCs	cancer stem cells
DSP	digital spatial profiling
FFPE	formalin-fixed paraffin-embedded
ICIs	immune checkpoint inhibitors
IHC	immunohistochemical staining
LUAD	lung adenocarcinoma
NSCLC	non-small cell lung cancer
PVS	perivascular stroma
ROI	region of interest
rpS6	ribosomal protein S6
SC	stromal cell
TC	tumor cell
TME	tumor microenvironment
TSI	tumor–stromal interface
